# Minimising OHSS in women with PCOS

**DOI:** 10.3389/fendo.2025.1507857

**Published:** 2025-03-13

**Authors:** Sebastian Leathersich, Caitlin Roche, Roger Hart

**Affiliations:** ^1^ Department of Reproductive Medicine, King Edward Memorial Hospital, Subiaco, WA, Australia; ^2^ City Fertility Australia, Claremont, WA, Australia; ^3^ Fertility Specialists of Western Australia, Claremont, WA, Australia; ^4^ Dexeus Fertility, Dexeus University Hospital, Barcelona, Spain; ^5^ Division of Obstetrics and Gynaecology, The University of Western Australia, Crawley, WA, Australia

**Keywords:** ovarian hyperstimulation syndrome, polycystic ovary syndrome, *in vitro* fertilization, assisted reproductive technology, agonist trigger, progestin-primed ovarian stimulation, *in vitro* maturation

## Abstract

Ovarian hyperstimulation syndrome (OHSS) is a serious iatrogenic complication of ovarian stimulation during *in vitro* fertilisation (IVF) treatment and is associated with significant morbidity and a small risk of mortality. Women with polycystic ovary syndrome (PCOS) are at a substantially increased risk of developing OHSS compared to those without. This paper reviews the current evidence for strategies to mitigate the risk of OHSS in this patient population. In order to minimise the risk of OHSS, clinicians should identify patients at high risk prior to commencing treatment and provide adequate pre-treatment counselling regarding the risks and benefits of IVF treatment, as well as alternative treatment options. Strategies that can reduce the risk of OHSS include co-treatment with metformin in gonadotropin releasing hormone (GnRH) agonist cycles, use of GnRH antagonist or PPOS protocols, appropriate gonadotropin dosing, the use of a GnRH agonist trigger for oocyte maturation in antagonist or PPOS protocols, cryopreservation of all embryos with deferred frozen embryo transfer, and treatment with dopamine-agonists after oocyte collection. *In vitro* maturation (IVM) offers an alternative with no risk of OHSS, however currently has a lower cumulative live birth rate than conventional IVF. These strategies can prevent significant early and late OHSS in women with PCOS and should be used to optimise the safety of IVF for this high-risk population, striving for OHSS-free treatment for all patients undergoing IVF.

## 1 Introduction

Ovarian hyperstimulation syndrome (OHSS) is a potentially life-threatening iatrogenic complication of ovarian stimulation for assisted reproductive technology (ART) (1–4). It can vary in severity, carrying considerable morbidity and a small risk of mortality (1, 2). While the true incidence is difficult to determine due to variable reporting requirements and the lack of a strict consensus definition, moderate-to-severe OHSS is considered to be uncommon, occurring in ~1-5% of *in vitro* fertilisation (IVF) cycles (3). One of the groups at highest risk of developing OHSS is women with polycystic ovary syndrome (PCOS). This article will review the evidence for minimizing the risk of OHSS in this population.

### 1.1 Pathophysiology of OHSS

IVF typically involves ovarian stimulation with exogenous gonadotropins with the goal of achieving multifollicular development, followed by oocyte maturation prior to oocyte retrieval. OHSS most frequently occurs when ovarian stimulation results in an exaggerated ovarian response (2). The pathophysiology of OHSS is not completely understood (4), however the pivotal stimulus is usually exogenous human chorionic gonadotropin (hCG) administered for final follicular maturation (“triggering”) in the presence of multiple peri-ovulatory follicles, resulting in the formation of numerous corpora lutea (5). This leads to excessive ovarian production of vascular endothelial growth factor (VEGF) and other vasoactive-angiogenic substances, resulting in increased vascular permeability and a fluid shift from the intravascular to extravascular spaces (2, 5). This intravascular depletion and extravascular accumulation of fluid results in the typical signs, symptoms and complications of OHSS.

OHSS can be classified into four stages (mild, moderate, severe or critical) based on symptom severity and laboratory findings (3, 4). The clinical manifestations are primarily a consequence of extravasation of fluid, whilst ovarian enlargement can also contribute to symptoms (4). OHSS can also be classified as early or late depending on the timing of onset (4). Early OHSS usually begins 4-7 days after administration of the trigger, whereas late OHSS typically begins 9 days or more after the trigger (4). Late OHSS usually occurs in the setting of pregnancy following a fresh embryo transfer, and tends to be more severe and prolonged than early OHSS due to the ongoing stimulation of the corpora lutea by the rising hCG of pregnancy (4). Early OHSS is normally self-limiting with spontaneous resolution within a few days, but may persist for longer in serious cases or in the case of a fresh embryo transfer and subsequent pregnancy (1).

There are no internationally accepted consensus criteria to define the severity of OHSS, however the Royal College of Obstetricians and Gynaecologists (RCOG) guidelines are frequently used (**Table 1**). Mild OHSS is characterised by symptoms and mild signs alone, whilst moderate and severe OHSS are accompanied by laboratory or sonographic evidence of worsening disease and may require hospital admission for management (1, 4). Critical OHSS is characterised by significant end-organ dysfunction and can result in life-threatening complications, often requiring admission to a high dependency unit (HDU) or intensive care unit (ICU) for management (1, 4).

**Table 1:** RCOG classification of severity of OHSS (6).

Category	Features
Mild OHSS	Abdominal bloating Mild abdominal pain Ovarian size usually < 8 cm
Moderate OHSS	Moderate abdominal pain Nausea ± vomiting Ultrasound evidence of ascites Ovarian size usually 8–12 cm
Severe OHSS	Clinical ascites (± hydrothorax) Oliguria (< 300 ml/day or < 30 ml/hour) Haematocrit > 0.45 Hyponatraemia (sodium < 135 mmol/l) Hypo-osmolality (osmolality < 282 mOsm/kg) Hyperkalaemia (potassium > 5 mmol/l) Hypoproteinaemia (serum albumin < 35 g/l) Ovarian size usually > 12 cm
Critical OHSS	Tense ascites/large hydrothorax Haematocrit > 0.55 White cell count > 25 000/ml Oliguria/anuria Thromboembolism Acute respiratory distress syndrome

### 1.2 Epidemiology of OHSS in patients with PCOS

OHSS may affect any patient undergoing ovarian stimulation, however patients at particular risk include younger patients, those with low body weight, previous OHSS, high ovarian reserve as indicated by elevated antral follicle count (AFC) or serum anti-mullerian hormone (AMH) levels, and patients with PCOS (1).

Whilst OHSS has become less frequent among patients with PCOS with the introduction of new techniques for ovarian stimulation, the true incidence remains uncertain, and it is likely under-reported (7). This is due to the lack of strict, objective diagnostic criteria for OHSS, the overlap between the symptoms of mild to moderate OHSS and other complications following transvaginal oocyte collection, and the lack of systematic data collection.

Overall, the incidence of OHSS (mild, moderate and severe) in patients with PCOS has been estimated to be as high as 17-31%, compared to an approximately 5% risk of moderate OHSS in the general population (8–10). However, these figures are based on older ART treatment approaches, including the exclusive use of hCG for triggering oocyte maturation, and may not reflect current practices (8–10). Population level data suggests a reduction in the incidence of OHSS from 2004 to 2007, with a plateau in the years since at approximately 1.5 hospital admissions per 1,000 IVF cycles in the United States (US) (11). Meanwhile, US Emergency Department data suggests that in 2016, there were 6.1 emergency visits per 1,000 IVF cycles (12). HFEA data from 2020/21 report a low incidence of severe or critical OHSS in the UK, with fewer than 0.1% of cycles affected (13), whilst 0.4% of IVF cycles in Australia and New Zealand in 2019 were complicated by a hospital admission for OHSS (14). This demonstrates that, whilst OHSS incidence has substantially diminished over the last 20 years, it continues to affect women undergoing fertility treatment.

### 1.3 Impact

The medical complications of OHSS may be significant. Whilst mild and moderate OHSS can often be managed conservatively on an out-patient basis, severe and critical OHSS usually require hospital admission and in some cases intensive care. Complications can include thromboembolic disease, anuria with acute renal failure, respiratory failure, cardiac arrhythmias, disseminated intravascular coagulation, ascites, hydrothorax, pericardial effusion, sepsis and acute respiratory distress syndrome (4). Mortality due to OHSS is reported in the literature, however the incidence is impossible to estimate due to the lack of consistent reporting. It is generally accepted to be very rare (6).

In addition to the immediate health risks to the patient, large population studies have also reported an association between OHSS and adverse obstetric outcomes when fresh embryo transfer is performed, including an increased risk of low birth weight and pre-term delivery (15).

The cost to the health system of OHSS is uncertain, with the limited published estimates varying substantially depending on the country and type of management analyzed (e.g. outpatient vs inpatient). One cost-analysis of conservative (non-surgical) inpatient management of moderate-to-severe OHSS in a North American context estimated the treatment cost at US$10,099 per patient in 2013 (16), whilst in 2016 the cost of an emergency department visit with OHSS in the US was estimated to be US$5,601 (12). In contrast to this, an analysis in the same year in Spain estimated the cost of conservative medical treatment in moderate-to-severe OHSS to be US$570 per patient (17). A recent study using French insurance data to model costs estimated the cost of mild and moderate OHSS to be €235.42, and the cost of severe OHSS to be €1,391.72 per patient (18). This wide variation in estimates may reflect regional differences in management approaches and in health system costs. Whilst these estimates show that there is a significant healthcare cost of OHSS, no studies have considered the economic consequences of income loss and patient impact, and the true economic burden of OHSS remains unknown.

There is little research evaluating the patient experience of OHSS, whether mild, moderate or severe. The data that is available suggests that for women affected by OHSS, the symptoms can have significant physical and emotional impacts, even in mild OHSS, with women reportedly taking weeks, and sometimes months, off work due to physical symptoms (19). The delays to fertility treatment can also cause emotional distress for affected women, whilst any need for hospitalisation has been reported to cause family distress and anxiety (19, 20).

Taking these factors into account, preventing OHSS should be a key goal of the contemporary practice of IVF. In this paper, we will review the optimisation of high-risk patients prior to commencing treatment, the mitigation of risk throughout ovarian stimulation and triggering of final oocyte maturation, and the reduction of OHSS risk following oocyte collection. We will also review the role of emerging techniques in reproductive medicine, such as *in vitro* maturation, in achieving an OHSS-free clinic.

### 1.4 Risk factors for OHSS

Given the morbidity, potential mortality, cost and patient burden associated with OHSS, it is imperative that clinicians identify patients at increased risk in order to provide appropriate counselling and employ strategies to reduce the risk.

Many women at increased risk of OHSS can be identified prior to commencing ovarian stimulation, with PCOS being one of the strongest and most consistent risk factors. A 2010 database study found ovulatory disorders to be the strongest risk factor evaluated, associated with an adjusted odd ratio (aOR) of 2.01 for OHSS, whilst an earlier meta-analysis reported an OR of 6.8 for OHSS in women with polycystic ovarian morphology (21). A sub-analysis of a more recent randomised controlled trial (RCT) revealed more than double the incidence of severe OHSS in women with irregular menses compared to regular menses in both antagonist and agonist cycles (22).

In keeping with PCOS being a major risk factor for OHSS, elevated ovarian reserve markers predict an increased likelihood of OHSS. Serum AMH levels are consistently shown to predict the risk of OHSS, however proposed cut-offs to define high-risk patients have varied widely from 3.36ng/ml up to 10ng/ml (23–28). An increased antral follicle count (AFC) has also been associated with an increased risk of OHSS, with one prospective cohort study of over 1,000 women finding that the rate of moderate-to-severe OHSS increased from 2.2% with an AFC <24 to 8.6% with an AFC ≥24 (28–31).

Thresholds for these measures of ovarian reserve are difficult to define. Previous studies have been performed in heterogeneous populations, whilst the type of treatments also vary. Furthermore, there is variation between AMH assay results, whilst AFC increases with improving ultrasound technology. Overall, whilst it is not possible to define a specific threshold for ovarian reserve tests that reliably predicts OHSS in all populations undergoing all types of IVF cycles, it is clear that elevated ovarian reserve is a strong risk factor for OHSS.

Other baseline characteristics that are associated with an increased risk of OHSS include younger age, cause of infertility (tubal factor, ovulatory disorders or unexplained) and Black race, whilst lower BMI has been inconsistently associated with increased OHSS risk (32).

## 2 Prevention of OHSS prior to starting ovarian stimulation

For patients at high risk of OHSS, such as those with PCOS, there are various measures that can be implemented to reduce that risk prior to and during ovarian stimulation. These include the use of metformin, appropriate gonadotropin dosing and selection of treatment cycle type, and the choice of trigger for oocyte maturation.

### 2.1 Metformin

Metformin is an insulin‐sensitising agent that has been shown to reduce the risk of OHSS in women with PCOS under certain circumstances, however its principal mechanisms of action remain uncertain (3, 33). Laboratory and animal studies have shown that metformin may reduce vasoactive factors including VEGF, angiopoietins, cyclooxygenase-2 (COX-2) and nitric oxide synthase (NOS), potentially reducing the vascular alterations underlying the pathogenesis of OHSS (34, 35). It has also been suggested that treatment with metformin reduces the number of non-periovulatory follicles on the day of trigger, as well as the peak estradiol level per periovulatory follicle (33, 36, 37).

There is substantial clinical evidence that metformin prior to or during ovarian stimulation decreases the risk of OHSS in women with PCOS undergoing IVF in a GnRH agonist protocol (37). This is summarised in a 2020 systematic review, which found that metformin may reduce the incidence of OHSS by more than half when compared with placebo or no treatment in patients undergoing a long GnRH agonist protocol (relative risk (RR) 0.40, 95% confidence interval (CI) 0.26 to 0.60) (37).

However, the effect of metformin on OHSS incidence when using GnRH antagonist protocols remains uncertain, with the only two randomised controlled trials reporting no difference in the rates of OHSS (37–39). Only one of these reported on clinical pregnancy outcomes, and found a significant reduction in clinical pregnancy rates and live birth rates (LBR) in the metformin group when compared to placebo (RR for LBR 0.48, 95% CI 0.29 to 0.79), although it was not powered for this outcome (39). In addition to these prospective data, retrospective studies have also failed to demonstrate a benefit of metformin in GnRH antagonist cycles, making its routine use for the prevention of OHSS unjustified in this context. There is no evidence regarding the impact of metformin treatment in conjunction with other cycle types, including in progestin-primed ovarian stimulation (PPOS).

Overall, for patients in whom an agonist-cycle is planned, pre-treatment with metformin reduces the incidence of OHSS, however in antagonist cycles there is no evidence of benefit and a possibility of harm. For patients undergoing PPOS cycles, there is no evidence to support or refute the use of metformin.

### 2.2 Pre-treatments other than metformin

Various other pre-treatments have been proposed to reduce the risk of OHSS, including myoinositol, D-chiro-inositol, and vitamin D, however there are no high-quality data supporting their use for this indication. One RCT of 102 women with PCOS compared myoinositol to metformin in GnRH antagonist cycles and reported no difference in the rates of OHSS, though confidence intervals were very wide (40). Similarly, a small RCT randomized 60 women with PCOS to myoinositol and two different doses of D-chiro-inositol and reported lower rates of OHSS in the group receiving the higher dose (3.4% vs 18.5%), though the difference was not statistically significant, and OHSS was a secondary outcome in this study (41).

Currently available evidence does not support the routine use of these medications for the prevention of OHSS in women with PCOS, and further high-quality studies are required.

## 3 Approaches to ovarian stimulation to reduce the risk of OHSS

### 3.1 Stimulation protocol

#### 3.1.1 GnRH antagonist versus GnRH agonist protocol

Since their introduction, the use of GnRH antagonists to prevent a premature luteinising hormone (LH) surge during IVF has become standard practice to minimise the risk of OHSS, with substantial evidence supporting this practice. Though both GnRH agonists and GnRH antagonists suppress pituitary function, their mechanisms of action are distinct. GnRH agonists bind and activate the GnRH receptor, resulting in an initial flare in gonadotropin secretion followed by internalisation and down-regulation of the receptor, whilst GnRH antagonists competitively and reversibly bind and inhibit receptor activation (42, 43).

For a general population of patients undergoing IVF, antagonist protocols have been shown to have comparable live-birth rates to agonist protocols with a significantly lower risk of OHSS, with a 2016 Cochrane Review reporting an odds ratio for OHSS of 0.61 (95% CI 0.51 to 0.72) in antagonist cycles compared to agonist cycles (42). A subsequent large, well-designed RCT randomised 1,050 infertile women to GnRH antagonist or GnRH agonist protocols in their first IVF cycle with an hCG trigger in all but 3 cases, and confirmed a significant reduction in the rate of severe and moderate OHSS (5.1% vs 8.9% and 10.2% vs 15.6% respectively) when an antagonist cycle was used, with no difference in subsequent live birth rates (22).

Specifically considering patients with a diagnosis of PCOS, a recent meta-analysis of 10 RCTs reported that, compared to GnRH agonist protocols, GnRH antagonist protocols are associated with a reduced risk of OHSS overall (OR 0.58, 95% CI 0.44 to 0.77), and specifically of moderate to severe OHSS (OR 0.65, 95% CI 0.51 to 0.82) (44). No difference was detected in the rates of ongoing pregnancy across these studies; a single study reported on live birth rates, also finding no difference (44).

Overall, the data demonstrate that, in women with PCOS, GnRH antagonist cycles are associated with a lower rate of moderate to severe OHSS than GnRH agonist cycles, even when an hCG trigger is used, and achieve comparable pregnancy rates. Given the substantial evidence for improved safety and equal efficacy, current international evidence-based guidelines recommend the use of GnRH antagonist protocols as the preferred approach to controlled ovarian stimulation for patients with PCOS (45, 46).

#### 3.1.2 Progestogen-primed ovarian stimulation

More recently, progestogen-primed ovarian stimulation protocols have entered clinical practice as an alternative to GnRH analogues to prevent the LH surge during ovarian stimulation (47). A recent Cochrane review, based on 14 RCT’s including 3,224 patients, evaluated the efficacy and safety of PPOS compared to GnRH analogues (48). Though the findings were of very-low to low certainty, the authors concluded there is likely little to no difference in live birth rates, and no cases of moderate or severe OHSS were reported in any of the included studies (48). Notably, this review did not include any studies specifically evaluating the use of PPOS in women with PCOS, and its role in this population remains uncertain (48).

Two recent, small meta-analyses have evaluated the efficacy of PPOS protocols compared to GnRH antagonist protocols specifically in women with PCOS, however included both randomised and non-randomised studies of generally low quality (49, 50). The first found no significant difference in the incidence of moderate or severe OHSS (49), whilst the second reported that PPOS was associated with a lower risk of OHSS compared with a GnRH antagonist protocol (50). Neither study found any difference in pregnancy outcomes between the two groups.

Subsequent to these meta-analyses, a recent RCT included 784 women with an anticipated high response to ovarian stimulation (AFC > 15) but not specifically with PCOS and randomised them to PPOS or an antagonist protocol, reporting no cases of moderate-to-severe OHSS in either group (51). They also found that PPOS was non-inferior in terms of live birth rate following first frozen embryo transfer and cumulative live birth rate within 6 months (51).

PPOS may have some advantages over conventional antagonist protocols, being cheaper and reducing the injection burden for patients, possibly leading to improved patient compliance (49). Furthermore, recent prospective and retrospective studies have suggested that the degree of pituitary suppression may be less in PPOS cycles compared to antagonist-controlled cycles, resulting in higher LH levels on the day of trigger (52). Whilst it remains to be evaluated in clinical studies, it is plausible that this could further reduce the already low risk of agonist trigger failure, further ensuring patient safety. It is also important to consider that PPOS protocols require all embryos be cryopreserved due to premature decidualisation of the endometrium with resultant endometrial-embryonic asynchrony (48, 49, 53).

Overall, PPOS appears to be an effective and safe alternative to the GnRH antagonist protocol in patients with PCOS, resulting in similarly low rates of moderate-to-severe OHSS without compromising clinical outcomes. There is, however, a need for well-designed prospective, randomised controlled trials evaluating its role specifically in patients with PCOS and including OHSS as well as cumulative live birth rates as outcomes.

#### 3.1.3 Minimal stimulation protocols

Minimal stimulation protocols aim to limit the number of developing follicles through restricting the maximum daily gonadotropin dose to 150IU of follicle stimulating hormone (FSH), or in some cases using oral medications such as clomiphene citrate (54). Whilst mild-dose IVF was associated with reduced OHSS rates in normal and hyper-responders in a recent meta-analysis, this analysis specifically excluded patients with PCOS (55). A retrospective study of 235 patients undergoing minimal stimulation in an agonist-protocol using an hCG trigger still reported moderate or severe OHSS in 16.9% of participants with PCOS, with 1.9% requiring hospitalisation (56). Meanwhile, a recent pilot randomised controlled trial used 100 to 150 IU/d of FSH or human menopausal gonadotropin (HMG) for ovarian stimulation in antagonist cycles with a hCG trigger in women with PCOS (57). This trial reported that 70.1% of participants required unplanned conversion to a freeze-all cycle due to a high risk of OHSS, seriously questioning the benefits of this approach in PCOS patients for the purpose of OHSS risk reduction over other treatment options (57).

In summary, for patients with PCOS who are at risk of OHSS, the conventional stimulation protocol of choice should be either a GnRH antagonist or PPOS protocol, both of which also permit the use of a GnRH agonist trigger. If fresh embryo transfer is to be considered, then a GnRH antagonist protocol is preferred. Minimal stimulation can be considered but should not replace the use of a GnRH antagonist or PPOS protocol.

#### 3.1.4 Gonadotropin dose

Selecting an appropriate gonadotropin starting dose and altering the dose during stimulation aims to achieve an “optimal” number of oocytes, avoiding either hypo- or hyper-response. Achieving an appropriate balance is important given that the number of oocytes retrieved is a strong predictor of both OHSS (15) and cumulative live birth rates (58).

Whilst there is moderate evidence from multiple RCTs and meta-analyses that individualised gonadotropin dosing based on ovarian reserve testing (by AMH level) results in a reduced risk of moderate and severe OHSS, all of these studies specifically excluded patients with PCOS (59–62). The well-designed OPTIMIST trial did include a group of patients predicted to be hyper-responders (AFC > 15) and found that, compared to a “standard” starting dose of 150IU of FSH per day, 100IU of FSH per day was associated with similar cumulative live birth rate (CLBR) and a lower rate of any grade of OHSS, though no difference in the rate of severe OHSS (61). Notably, this study specifically excluded patients with PCOS, and used a GnRH agonist protocol in over 70% of cases, limiting the applicability of the data to the contemporary practice of IVF in patients with PCOS (61).

Overall, initial gonadotropin dose selection in patients with PCOS can be challenging, and needs to balance the desire to avoid both hypo- and hyper-response. Alongside the substantial evidence of an increased risk of hyper-response and OHSS in patients with PCOS, there is also evidence that they may display gonadotropin resistance, requiring higher doses to achieve the desired follicular development (63). These challenges are compounded by the fact that there is no high-quality evidence guiding the starting dose of FSH for ovarian stimulation in this population.

In practice, it is not uncommon for clinicians to tailor the starting dose of FSH during controlled ovarian stimulation based on patient characteristics such as age, previous response to stimulation and presence of PCOS (64). While more research is needed in this area, an evidence-based approach to tailoring the FSH dose could increase the likelihood of an appropriate ovarian response, thereby increasing cumulative pregnancy and live birth rates, whilst also reducing the risk of OHSS and cycle cancellation (for both poor and hyper-responders) (64). There is ongoing research into the role of artificial intelligence in this area, and the ability to combine large volumes of data may provide the information needed to guide gonadotropin dosing for patients in the future (65).

## 4 Reducing the risk of OHSS during ovarian stimulation

### 4.1 Dose adjustment

In addition to individualisation of the starting gonadotropin dose, doses can be adjusted during the stimulation cycle depending on patient response. Whilst evidence suggests that dose adjustment is performed frequently in clinical practice, there is no high quality evidence regarding the effect of intra-cycle dose adjustment on the risk of OHSS in patients with PCOS (66).

### 4.2 Coasting

For patients who have an exaggerated response to ovarian stimulation and are at risk of OHSS, “coasting” has been proposed as a means to reduce the risk of OHSS. This involves withholding gonadotropin doses and allowing estradiol levels to fall prior to administering the trigger for final oocyte maturation (3, 5).

A meta-analysis of two RCTs that included patients with a previous history of OHSS who were undergoing ovarian stimulation in GnRH agonist protocols found low quality evidence that coasting was associated with a reduction in OHSS. However, methodological concerns regarding the included studies and the included patient populations as well as the use of GnRH agonist protocols limit the applicability of these findings to the contemporary management of patients with PCOS (67). Furthermore, some cohort studies have reported a reduction in implantation rates after 4 or more days of coasting, questioning the suitability of this approach if a fresh embryo transfer is planned (68).

Overall, coasting may offer an option to reduce the risk of OHSS in settings where alternative, more strongly evidence-based, options to minimise the risk are not available, however should not be considered a first-line strategy.

### 4.3 Cycle cancellation

In patients who are at an unacceptably high risk of OHSS, cycle cancellation is an option to avoid OHSS (5, 69). By withholding an hCG trigger, prolonged stimulation of the corpora lutea is avoided and the physiological changes that lead to significant OHSS are abrogated (69). However, cycle cancellation also results in wasted resources, financial implications and both emotional and psychological distress for patients, and may also result in the cancellation of cycles that would not have progressed to clinical OHSS (5, 70). Whilst cancellation remains a physiologically justifiable option to prevent OHSS in certain settings, there are no clinical studies evaluating its efficacy (69).

Cycle cancellation is rarely indicated when an agonist trigger is possible, however for patients with an unexpectedly high response in agonist-controlled cycles requiring hCG triggering it should be considered to mitigate the risk of moderate to severe OHSS.

## 5 Reducing OHSS risk around the time of triggering final oocyte maturation

### 5.1 GnRH agonist trigger

The final step in conventional ovarian stimulation prior to oocyte collection is the triggering of oocyte maturation, which is achieved by activation of the LH/hCG receptor by one of its ligands. Historically, the standard trigger was recombinant or urinary hCG, which remains the only option in GnRH agonist protocols. However, with the advent of GnRH antagonist protocols and PPOS, oocyte maturation can also be achieved by administering a GnRH agonist, which results in an endogenous gonadotropin surge through stimulation of the pituitary GnRH receptor.

Whilst both hCG and GnRH agonists can trigger oocyte maturation, they have very different pharmacokinetic properties, making the use of hCG as a trigger one of the main risk factors for developing OHSS. The natural LH surge is characterised by an ascending phase of 14 hours, a plateau of 14 hours, and a 20-hour descending phase, whilst GnRH agonist administration results in a 4-hour ascending phase followed by a 20-hour descending phase (71). This relatively short duration of action results in sufficient LH/hCG receptor stimulation to effect nuclear and cytoplasmic oocyte maturation, while avoiding ongoing stimulation of the corpora lutea (71). In contrast to this, hCG has a half-life of approximately 34 hours, resulting in prolonged stimulation of the corpora lutea after oocyte collection and increasing the risk of OHSS (71).

A meta-analysis of 17 RCTs involving 1847 patients compared the effectiveness and safety of GnRH agonists to hCG for triggering final oocyte maturation in GnRH antagonist protocols (72). They reported that in donor-recipient cycles a GnRH agonist trigger resulted in a lower incidence of OHSS in donors when compared to an hCG trigger, with no difference in recipient live birth rates (72). The remaining thirteen studies assessed fresh autologous cycles, again reporting a lower incidence of mild, moderate or severe OHSS with a GnRH agonist trigger when compared to hCG, but also a lower live birth rate (72). This effect on live birth rates is well characterised. The GnRH agonist-induced LH surge is relatively short, as noted above, and whilst adequate to achieve nuclear and cytoplasmic maturation of the oocyte, results in a luteal phase deficiency (73). This reduces the incidence of OHSS, but also results in low pregnancy rates when a fresh embryo transfer with standard luteal support is performed (74).

Importantly, when a freeze-all strategy is used, avoiding the defective luteal phase, the reproductive outcomes appear to be similar irrespective of whether a GnRH agonist or a combined GnRH agonist/hCG trigger is used, supporting the efficacy of agonist triggering in oocyte maturation (75).

Despite increased utilisation of freeze-only IVF protocols, multiple strategies have been proposed for modified luteal phase support to allow fresh embryo transfer following GnRH agonist triggering (74). These modifications have included intensive exogeneous luteal phase steroid support with estradiol and progesterone, a “dual trigger” with a GnRH agonist combined with a reduced dose of hCG, an hCG bolus at the time of oocyte retrieval, or luteal phase hCG or GnRH agonist administration (10, 76–82).

OHSS has been reported following exposure to even low doses of hCG, whether at the time of trigger or after oocyte collection, as has late OHSS following embryo transfer after a GnRH agonist trigger (77, 83, 84). Furthermore, there are no RCTs comparing the CLBR following GnRH agonist trigger with intensive luteal support and a fresh embryo transfer to GnRH agonist trigger and a freeze-all approach with deferred frozen embryo transfer, which would be the ideal comparison. Overall, given the likely benefit of deferred embryo transfer in terms of live birth rate and obstetrical outcomes, and the comparable live birth rates, the role of “luteal phase rescue” in the context of an efficient cryopreservation program remains to be proven.

In summary, GnRH agonist triggers can reduce the risk of moderate to severe OHSS substantially, and in the absence of additional luteal phase support may well eliminate severe OHSS (85). However, even with an agonist-only trigger and no subsequent luteal phase support, mild or even moderate OHSS can still occur, and both patients and clinicians should be aware of this (75).

### 5.2 Dopamine agonist at the time of trigger

While the pathophysiology of OHSS is not completely understood, it is known that VEGF contributes to the increased vascular permeability and fluid shifts that characterise the more serious manifestations of OHSS (86). Dopamine agonists are capable of selectively inhibiting the increased vascular permeability caused by VEGF, thereby reducing the incidence of OHSS without interfering with the outcome of IVF cycles (86). While the mechanism is not completely clarified, it is thought to be due to reduced phosphorylation and therefore reduced activation of the VEGF receptor (VEGFR-2) (86).

A recent Cochrane review published in 2021 included 22 RCTs involving a mixed population of 3171 women at high risk of OHSS, with one study including only women with PCOS and a further 18 including women both with and without PCOS (87). Of the included studies, only two used an antagonist protocol (n = 162), one of which specifically excluded patients with PCOS (87). All studies used an hCG trigger with a fresh embryo transfer approach (87). The review found moderate-quality evidence that dopamine agonists, specifically cabergoline and quinagolide, probably reduce the risk of moderate or severe OHSS when compared to no intervention or placebo (87). The effect of dopamine agonists on live birth rates, clinical pregnancy, multiple pregnancy, miscarriage and adverse events remains uncertain due to very-low to low quality available evidence (87).

While there is no evidence of long-term complications associated with the low dose, short-duration treatment regimens used in ART to prevent OHSS, long-term use of cabergoline has been associated with cardiac valvulopathy in patients treated for prolactinomas and Parkinson’s disease, and the potential risks should be weighed against the benefits (86).

Overall, there is no good quality evidence that cabergoline or other dopamine agonists are of benefit in patients with PCOS undergoing ovarian stimulation in GnRH antagonist protocols with an agonist trigger, however in contexts where these treatment options are not possible, administration of an oral dopamine agonist from the time of hCG trigger or oocyte collection can reduce moderate-to-severe OHSS.

### 5.3 GnRH antagonist administration post-oocyte collection

GnRH antagonist administration after oocyte collection has been proposed as a potential strategy to prevent and treat OHSS. Possible mechanisms of action include inhibition of pituitary LH secretion and subsequent rapid luteolysis with a reduction in ovarian production of angiogenic factors, or possible direct effects via ovarian GnRH receptors, with GnRH antagonist administration having been shown to reduce circulating VEGF levels (88–92).

There are, however, no high-quality data supporting the use of GnRH antagonists for the prevention of OHSS. A single randomised controlled trial of 48 women (39 of whom had PCOS) undergoing ovarian stimulation in a GnRH agonist protocol with an hCG trigger and a plan to freeze all embryos compared 3 days of cetrorelix following oocyte collection to supportive treatment alone (93). There was no difference in the rates of moderate-to-severe OHSS, however the group treated with cetrorelix had lower estradiol levels and lower pain scores over the week following oocyte collection (93).

Various prospective cohort studies have been published involving diverse patient groups and variable stimulation protocols, and deliver conflicting results, with no clear benefit of luteal phase GnRH antagonists demonstrated with regard to the incidence of OHSS (94, 95).

Given the lack of high-quality evidence, the role of GnRH antagonists in the luteal phase for the prevention of OHSS is uncertain, and they should not be used as an isolated strategy to prevent moderate-to-severe OHSS. Whether certain patient populations may benefit remains to be determined, however there may be some utility in patients with unexpected hyper-response or an unexpectedly high number of oocytes retrieved as part of a multimodal management strategy.

## 6 Cycle segmentation to reduce the risk of OHSS

### 6.1 Freeze-all approach

Avoiding a fresh embryo transfer through cryopreservation of all embryos (“freeze-all”) reduces the incidence of OHSS in cycles at high risk, including for women with PCOS. It avoids the risk of late OHSS that comes with increasing levels of endogenous hCG and allows the use of a GnRH agonist trigger without additional luteal phase support. Furthermore, a planned frozen, rather than fresh, embryo transfer may also be associated with increased live birth rates and improved obstetric and neonatal outcomes compared with fresh embryo transfers in hyperstimulated cycles, further improving the safety and long-term outcomes of IVF (96, 97).

In the largest RCT to date, 1508 women with PCOS undergoing their first IVF cycle were randomized to either fresh embryo transfer or a freeze-all cycle with subsequent frozen embryo transfer (96). All patients underwent ovarian stimulation in a GnRH antagonist cycle with an hCG trigger and had up to two day 3 embryos transferred; patients at high risk of OHSS were excluded due to one of the arms having a fresh transfer (96). The study reported a significantly higher live birth rate after first frozen embryo transfer compared to fresh embryo transfer (49.3% vs. 42.0%; RR 1.17, 95% CI 1.05 to 1.31), along with a significant reduction in the incidence of moderate or severe OHSS (1.3% vs. 7.1%; RR 0.19, 95% CI 0.10 to 0.37) for frozen *vs* fresh transfers respectively, with comparable cumulative live birth rates over the ensuing 12 months (96). There was a higher incidence of pre-eclampsia in the frozen embryo transfer group (4.4% vs 1.4%), however no included patients developed severe pre-eclampsia (96). The frozen embryo transfers in this study were performed in artificial cycles with exogenous estrogen and progestogen, which is a known risk factor for pre-eclampsia and may explain this difference (98).

In a subsequent meta-analysis comparing the outcomes of the first embryo transfer using either a freeze-all or fresh transfer strategy, the above study was the only identified study including high-responding patients that adequately reported on OHSS risk, giving the same result (99). When combined with data from two smaller studies on high responders (though not specifically those with PCOS), a similar increase in LBR was seen with frozen embryo transfer compared to fresh (RR 1.18, 95% CI 1.06 to 1.31) (99).

Overall, there is high quality evidence showing that a freeze-all approach with deferred cryopreserved embryo transfer significantly reduces the likelihood of moderate-to-severe OHSS in women with PCOS undergoing IVF, whilst increasing the live birth rate per first embryo transfer. The apparent increase in pre-eclampsia risk may be associated with endometrial preparation techniques, and use of a natural or modified natural transfer cycle could reduce this risk (98). Planned cryopreservation of all embryos also allows clinicians to administer a GnRH agonist trigger, further minimising the risk of OHSS and allowing clinics to achieve very low rates of moderate-to-severe OHSS in these high risk patients (3, 99).

## 7 *In vitro* maturation

Whilst contemporary ovarian stimulation with agonist-triggering and cycle segmentation with a freeze-all approach significantly reduces the rate of OHSS, the condition still occurs in a small number of patients. Some studies have reported mild to moderate OHSS in up to 38% of high responders receiving an agonist trigger, though with no cases of severe OHSS (100). Furthermore, the process of ovarian stimulation is not without burden even in the absence of OHSS, particularly for hyper-responders. It involves multiple injections, substantially elevated hormone levels, ovarian enlargement and discomfort, and the risk of ovarian torsion.

Recent advances in the efficacy of *in vitro* maturation (IVM) in humans has provided an alternative option for assisted reproduction in patients with PCOS which offers few or no injections, hormone levels within the physiological range for a normal menstrual cycle, and a reported zero risk of OHSS due to the lack of ovarian stimulation and the absence of a trigger for oocyte maturation.

The principle of IVM is to collect immature oocytes from the antral follicles of unstimulated or minimally stimulated ovaries, with maturation to metaphase II stage occurring in the laboratory prior to fertilisation or vitrification. There exist various approaches to clinical IVM, which differ in the use of ovarian stimulation, the use of a trigger, and the laboratory approaches. This paper will not consider IVM with a trigger or “rescue” IVM when immature oocytes are retrieved during a conventional oocyte collection.

### 7.1 Different approaches to IVM

#### 7.1.1 Monophasic versus biphasic IVM

Most, if not all, of the success of IVM depends on laboratory factors. The two main laboratory approaches to IVM at present are monophasic or biphasic IVM, also known as capacitation IVM or CAPA-IVM. The latter includes an additional pre-maturation phase in the laboratory, adding C-type natriuretic peptic (CNP) in order to prevent the spontaneous resumption of meiosis during IVM culture (101). It has been suggested that delaying the nuclear maturation of oocytes in this way can enhance the synchronicity of cytoplasmic and nuclear maturation, enhancing oocyte competence (102, 103).

A clinical trial of CAPA-IVM compared to monophasic IVM among 80 women with PCOS or polycystic ovarian morphology (PCOM) reported a significantly higher clinical pregnancy rate per day 3 embryo transfer of 63.2% in the CAPA-IVM group, compared to 38.5% in the monophasic IVM group (103). Subsequent data from the same group reported a non-significant difference in LBR per embryo transfer of 50.0% for CAPA-IVM compared to 33.3% for standard IVM (103), whilst a recent retrospective analysis of 1,563 IVM cycles in predicted hyper-responders (i.e. PCOS or a high AFC) reported cumulative live birth rates of 37.8% per commenced CAPA-IVM cycle (104).

As with all novel procedures and technologies, there is a learning curve for clinicians and embryologists when implementing IVM into clinical practice. Although not confirmed by rigorous data, it is very likely that IVM success rates will relate to clinic volume and laboratory experience, with international guidelines recommending that the clinical use of IVM should be restricted to units with sufficient expertise (45).

#### 7.1.2 Stimulated versus unstimulated IVM

It remains debated whether ovarian “priming” with 2-5 days of a moderate dose of FSH (e.g. 150 IU/d) should be administered prior to oocyte collection for IVM.

Recent randomised, controlled trial data in a Vietnamese population of women with PCOS aged ≤37 has demonstrated that, when using CAPA-IVM, there was no difference in the number of mature oocytes retrieved with or without FSH priming. Though not powered for the outcome of live birth, there were no significant between group differences (105). Whilst the restricted population of predominantly lean women with PCOS may limit the broader applicability of these findings, overall, it does not seem that FSH administration prior to oocyte collection is necessary or advantageous in IVM.

### 7.2 Outcomes of IVM compared to IVF

A non-inferiority RCT of 351 women with PCOS between the ages of 20 and 38 compared monophasic IVM without ovarian stimulation to a GnRH-antagonist cycle, with a freeze-all approach and deferred single blastocyst transfer in both groups (106). The study found that the ongoing pregnancy rate leading to live birth was significantly lower in the IVM group (22.3%) than in the conventional IVF group (50.6%) (106). No patients in the IVM group developed moderate or severe OHSS, whilst in the conventional IVF group moderate OHSS occurred in 5.7% of patients and severe OHSS in 0.6% (106). Importantly, this study used an hCG trigger in the conventional ovarian stimulation group, which does not reflect contemporary practice for freeze-all cycles in hyper responders.

An RCT comparing CAPA-IVM with FSH priming to conventional IVF in women with a high antral follicle count (≥24) reported a live birth rate of 35.2% (107). However, this study did not confirm non-inferiority of CAPA-IVM compared to IVF, with live birth rates in the conventional IVF group of 43.2% (107). Long-term follow up of 273 women in the RCT group reported a cumulative live birth rate of 44.0% over 12 months following CAPA-IVM (107). These results are supported by retrospective analyses from the same group. A retrospective analysis of all women with PCOS or a high AFC undergoing CAPA-IVM over a three-year period (n = 374) found that 98.4% of women had at least one day 3 embryo for transfer, and the cumulative live birth rate at 24 months after starting CAPA-IVM treatment was 38.5% (108).

Meanwhile, a retrospective analysis of 1,563 CAPA-IVM cycles from a centre with extensive IVM experience reported a cumulative live birth rate per cycle of 37.8% in a patient population with a mean age of 29.6 and a predicted hyper response to ovarian stimulation (104).

Whilst RCT data have not demonstrated non-inferiority of IVM compared to conventional IVF, a recent retrospective cohort study of first cycles in predicted hyper-responding patients (AMH ≥3.25 ng/ml) undergoing either highly purified hMG (HP-hMG) primed IVM (n = 463) or conventional IVF in an antagonist protocol (n = 1,244) has raised the possibility that the outcomes may depend on ovarian reserve (109). Overall, the authors found that IVM was associated with a lower cumulative ongoing pregnancy rate (OPR) per commenced cycle (42.8% compared to 63.8%), as well as lower fertilisation rates and a greater probability of not having any embryos available for transfer (13.6% vs 5.5%) (109). Taking into account covariates, a multivariable regression analysis reported an odds ratio for cumulative OPR of 3.73 (95% CI 2.64 to 5.26) for conventional ovarian stimulation compared to IVM (109). However, in a subgroup analysis the cumulative OPR and LBR were observed to converge as AMH increased, with no significant difference between treatment groups in patients with an AMH level >10 ng/ml (CLBR 46.7% in the IVM group compared to 59.6% in the conventional IVF group) (109).

There is also uncertainty whether women with specific PCOS phenotypes may achieve different results with IVM. Though there are no prospective studies evaluating this, a retrospective analysis of 320 women reported an increased cumulative live birth rate following the first IVM treatment cycle for women with PCOS phenotype A (hyperandrogenism + ovulatory disorder + polycystic ovaries), compared to those with phenotypes C (hyperandrogenism + polycystic ovaries) or D (ovulatory disorder + polycystic ovaries) after multivariable logistic regression analysis (110).

Taken together, the data suggest that the overall efficiency of IVM in patients with PCOS or elevated AMH levels remains less than that of conventional IVF with regard to pregnancy rates and live birth rates. However, in patients with very elevated AMH levels, the differences in outcomes appear to be abrogated and ongoing pregnancy rates are similar, whilst OHSS does not occur in patients undergoing IVM, suggesting that this patient population may benefit most from IVM.

### 7.3 Fresh or frozen transfer in IVM

Successful live birth has been reported with fresh embryo transfer following IVM, however the challenges presented by endometrial-embryo asynchrony have made it a less common approach (111). Indeed, a RCT comparing fresh embryo transfer to a freeze-all strategy in CAPA-IVM reported live birth rates of 20% and 60% respectively, suggesting that currently a freeze-all strategy should be routine when performing IVM (112).

Given the results of clinical studies and the lower live birth rates and ongoing pregnancy rates with IVM compared to conventional ovarian stimulation, the main benefit from IVM lies in the reduced patient burden. Indeed, Braam et al. reported that women in the Netherlands were willing to accept a reduction in pregnancy rates in order to reduce the risk of OHSS (113). It is important to note that the Netherlands offers fully funded infertility treatment, and this willingness to accept a reduction in clinical success rates has not been demonstrated in settings where patients are required to pay out-of-pocket for treatment.

Overall, IVM offers a low risk, lower-intervention, effective and cost-effective option for fertility treatment in selected patients with PCOS, with no risk of OHSS (**Table 2**). However, at present evidence suggests that clinical pregnancy and live birth rates are lower compared to conventional IVF/ICSI. Implementation of IVM into clinical practice also requires a laboratory with proven competence and efficiency in IVM, necessitating additional training. Though currently offered in limited centres, ongoing developments to improve the efficiency and efficacy of IVM may see it become the standard of care for patients with PCOS.

**Table 2:** Advantages and disadvantages of IVM compared to conventional IVF for women with PCOS.

Advantages	Disadvantages
Reduced duration of treatment	Reduced efficiency
Reduced drug use and cost	Not suitable for all patients
No risk of OHSS	Increased oocyte collection time

## 8 Future research considerations

As identified by this review, despite significant advances in the prevention of OHSS over recent decades there remain several substantial gaps in the literature which are relevant for the optimization of fertility treatment in women with PCOS and the avoidance of OHSS.

The first is the uncertainty regarding the incidence and severity of OHSS in this population due to a lack of objective diagnostic and severity criteria and unreliable reporting. Without systematic, national data collection, monitoring of IVF outcomes and complications is severely impaired, and reporting on OHSS incidence and severity should be a requirement of all fertility clinics.

Secondly, there is a lack of high-quality data on the impact of OHSS on patients, including the physical, psychological, social and economic burden. There is also little evidence on the impact that developing OHSS has on future reproductive decision making, including the likelihood of pursuing further fertility treatment, which could ultimately affect the likelihood of achieving a live birth.

There are also shortcomings in the available evidence regarding therapeutic options available to women with PCOS to enhance the safety of IVF and minimize the risk of OHSS. For example, whilst insulin-sensitizing treatments other than metformin (such as myoinositol and d-chiro-inositol) are widely used, they remain understudied in women with PCOS, whilst the role of metformin itself in GnRH antagonist or PPOS cycles is also uncertain.

Similarly, the use of progestogens for pituitary suppression (PPOS) has become increasingly popular in freeze-all cycles, and a growing body of evidence supports its efficacy and safety when compared to GnRH antagonist cycles. However, to date there are no well-designed, prospective, randomized clinical trials evaluating PPOS specifically in patients with PCOS, and the justification for its use has been extrapolated from non-randomized studies or RCTs in women with a high AFC (>15) but not necessarily PCOS.

As with all areas of medicine, artificial intelligence has the potential to alter the practice of reproductive medicine. Aside from a role in the laboratory, recent studies have demonstrated that AI could assist with gonadotropin dose selection and adjustment as well as timing of triggering in ovarian stimulation (114). Given the multiple variables and the large amount of data that must be integrated, the applications of machine learning for individualizing ovarian stimulation in women with PCOS cannot be overlooked, potentially helping to optimize mature oocyte yield and clinical outcomes whilst preventing OHSS. As AI is integrated into more clinics and patient and clinician acceptance increase, it will likely play a major role in clinical practice in coming years.

Looking forward, other novel treatments have the potential to expand fertility options for women with PCOS. For example, the growing interest in *in vitro* activation and growth of oocytes from primordial follicles could allow patients with PCOS, or other ovarian pathologies, to bypass not just ovarian stimulation (as in IVM), but oocyte collection altogether (115). This could offer the possibility of harvesting a small piece of ovarian cortex to create embryos *in vitro*.

It is also likely that glucagon-like peptide-1 (GLP-1) and glucose-dependent insulinotropic polypeptide (GIP) agonists will play a greater role in the management of PCOS as these medications become ever more widely used for weight loss and the management of metabolic syndrome and diabetes. The metabolic and endocrine benefits of these medications are substantial, and can improve many of the features of PCOS, including insulin resistance, hyperandrogenism and overweight and obesity (116). Whilst treatment with GLP-1 agonists has been shown to increase natural conception rates and improve menstrual cycle irregularity (117), the impact on IVF outcomes is uncertain. There are also concerns regarding the effects on an early pregnancy, and a washout period of several months is advocated prior to conception (118). Whether such treatments, through their impact on the metabolic features of PCOS, could reduce the risk of OHSS whilst improving the outcomes of IVF treatment remains to be tested in prospective clinical trials.

Other potential treatments have also been proposed, including intravenous calcium gluconate (119), kallistatin (120), coenzyme Q10 (121) and local N-acetylcysteine (122). At present these approaches remain experimental with data limited to *in vitro* or animal studies or small human studies, and further evidence is required before they can be recommended in clinical practice.

## 9 Conclusions

Women with PCOS are at high risk of OHSS, which can be associated with significant morbidity or even mortality. Patient counselling should include discussion of the risks and likely outcomes of treatment, including options to optimise these, and IVF should be reserved for patients in whom it is indicated. To reduce the risk of OHSS for women with PCOS undergoing IVF, GnRH antagonist protocols should be used over GnRH agonist protocols, with GnRH agonist triggering for final oocyte maturation. Progestin-primed ovarian stimulation with a GnRH agonist trigger may be a suitable option in patients at high risk of OHSS however high-quality evidence examining this outcome are lacking.

Appropriate dosing of gonadotropins, taking into account ovarian reserve testing and previous ovarian response, can also decrease the risk of OHSS. For women at high risk of OHSS who are undergoing ovarian stimulation in a GnRH agonist protocol, metformin may reduce the incidence of OHSS, however there is insufficient evidence to support its use in GnRH antagonist or PPOS cycles.

For patients at high risk of OHSS after triggering of final oocyte maturation, particularly with hCG, oral cabergoline can reduce the risk of developing OHSS, whilst there is also low-level evidence to support the use of GnRH antagonists after oocyte collection. A freeze-all strategy should be employed in women at risk of OHSS in order to reduce OHSS rates and prevent late OHSS, as well as potentially improve reproductive outcomes.

Finally, IVM can offer an OHSS-free practice, though currently at the cost of reduced live birth rates per cycle. As IVM efficiency improves and more laboratories develop expertise in this area, its use may increase in this population.

OHSS is a serious complication of IVF, and women with PCOS are at particularly high risk. Currently available treatment options allow the prevention of significant early and late OHSS in the majority of patients and should be employed to optimise the safety of fertility treatment and continue to aim for OHSS-free treatment.
